# Manifestations of Toxic Shock Syndrome in Children, Columbus, Ohio, USA, 2010–2017^1^

**DOI:** 10.3201/eid2606.190783

**Published:** 2020-06

**Authors:** Aliza Cook, Sarah Janse, Joshua R. Watson, Guliz Erdem

**Affiliations:** The Ohio State University, Columbus, Ohio, USA (A. Cook, S. Janse, J.R. Watson, G. Erdem);; Nationwide Children’s Hospital, Columbus (S. Janse, J.R. Watson, G. Erdem)

**Keywords:** Toxic shock syndrome, streptococcal TSS, nonstreptococcal TSS, children, diagnostic criteria, clindamycin, IVIg, intravenous immunoglobulin, bacteria, Ohio, streptococci

## Abstract

Data are limited on the incidence and management of streptococcal toxic shock syndrome (TSS) and nonstreptococcal TSS in children. We aimed to define the clinical patterns of TSS at Nationwide Children’s Hospital in Ohio as they relate to published criteria, diagnostic decisions, and treatment options. Through retrospective chart reviews, we identified 58 patients with TSS (27 streptococcal, 31 nonstreptococcal) during January 2010–September 2017. We observed clinical and laboratory findings that are not part of TSS criteria, such as pyuria in streptococcal TSS (50% of patients) and pulmonary involvement (85%) and coagulopathy (92%) in nonstreptococcal TSS patients. Recommended treatment with clindamycin and intravenous immunoglobulin was delayed in streptococcal TSS patients without rash (3.37 days vs. 0.87 days in patients with rash), leading to prolonged hospitalization and complications. Incorporation of additional TSS signs and symptoms would be helpful in TSS diagnosis and management.

## Abstract 2

We found unexpected pulmonary, coagulation, and urinary abnormalities and, in children without rash, delayed treatment.

Toxic shock syndrome (TSS) is a severe acute illness caused by toxin-producing strains of *Streptococcus pyogenes* (streptococcal TSS [STSS]) and *Staphylococcus aureus* (nonstreptococcal TSS [NSTSS]) (*1*–*5*). Data are limited on the incidence, management, and outcomes for children with TSS (*6*–*8*). Most epidemiologic and clinical studies of TSS have reported predominantly streptococcal cases. The incidence of severe streptococcal infections, including TSS, has been increasing in North America since the 1990s. Studies from the United States and Canada report incidence rates of 3.8–10.24 invasive group A *Streptococcus* (GAS) cases per 100,000 persons per year (*9*,*10*). Reported death rates vary from 4.2% to 56% for STSS (*2*,*3*,*7*,*8*,*11*–*15*) and are up to 22% for NSTSS (*3*,*12*).

The uncontrolled cytokine stimulation by bacterial toxins results in several clinical manifestations of TSS, such as hypotension, fever, rash, and organ dysfunction (*3*,*8*). On the basis of these clinical signs, the Centers for Disease Control and Prevention (CDC) defined criteria for TSS and updated these criteria in 2010 for STSS and in 2011 for NSTSS (*4*,*5*). Despite recognition of illness and CDC disease definitions, TSS diagnosis remains challenging because some clinical findings, such as hypotension, could be transient and some, such as rash, might be absent. 

CDC criteria do not differentiate between children and adults. In children, differentiating TSS from septic shock, Kawasaki disease with shock, and drug reaction with eosinophilia and systemic symptoms syndrome poses additional challenges (*3*,*16*). In addition to these diagnostic difficulties, challenges and controversies exist for managing TSS. Our objective was to differentiate the clinical patterns of TSS in children in a large tertiary center as they relate to published criteria, diagnostic decisions, and treatment options.

## Methods

The Nationwide Children’s Hospital Institutional Review Board approved the study. We queried electronic medical records during January 2010–December 2017 for diagnosis codes from the International Classification of Diseases, 10th Revision, for toxic shock syndrome, severe sepsis with septic shock, GAS bacteremia, and necrotizing fasciitis. The addition of diagnoses other than TSS helped us identify 7 STSS patients who were discharged with diagnoses of sepsis and septic shock. In addition to discharge and admission diagnoses, we considered any diagnosis during the hospitalization period associated with these billing codes. We also reviewed preadmission records related to the admission diagnoses. We included patients in the study if their illness met criteria for definite or probable TSS. Where all CDC criteria for either STSS or NSTSS were fulfilled, we defined cases as definite (*4*,*5*). Where a single criterion was missing because of incomplete data or because GAS was isolated from a nonsterile site, we classified the case as probable (*4*). Data collected were age, sex, height, weight, body mass index, date of presentation, and clinical signs and symptoms (Table 1). We also collected duration of hospitalization; use and type of antimicrobial agents; use and timing of clindamycin, intravenous immunoglobulin (IVIg), or both after initial evaluation; and clinical outcomes, including mechanical intubation, amputation, and death. We reviewed several admission laboratory and radiologic parameters: complete blood count, urinalysis, blood urea nitrogen, creatinine, estimated glomerular filtration rate, alanine transaminase, aspartate transaminase, total bilirubin, creatinine kinase, coagulation studies, and chest radiographs. We also reviewed additional studies and any imaging study done during the hospitalization period that identified possible sites and sources of infection.

**Table 1:** Characteristics of patients with STSS and NSTSS, Nationwide Children’s Hospital, Columbus, Ohio, USA, 2010–2017*

Characteristic†	STSS, n = 27	NSTSS, n = 31	Total, N = 58	p value
Demographic				
Sex, no. (%)				
F	15 (55.6)	21 (67.7)	36 (62.1)	0.42
M	12 (44.4)	10 (32.3)		
Mean age, y (SD)	9.4 (5.9)	13.2 (4.1)	11.4 (5.3)	<0.05
Mean BMI (SD)	18.9 (4.9)	22.7 (7.9)	20.9 (6.9)	<0.05
Clinical findings				
Vomiting	18 (66.7)	26 (83.9)	44 (75.9)	0.22
Diarrhea	9 (33.3)	14 (45.2)	23 (39.7)	0.43
Myalgia	9 (37.5)	12 (44.4)	21 (41.2)	0.78
Fever at presentation, mean (SD)	39.5 (0.8)	39.6 (0.6)	39.5 (0.7)	0.65
Generalized erythematous rash	14 (51.9)	30 (96.8)	44 (75.9)	<0.05
Desquamation	6 (22.2)	14 (45.2)	20 (34.5)	0.1
Altered mental status	10 (41.7)	11 (36.7)	21 (38.9)	0.83
Fasciitis/tissue necrosis	5 (20.0)	1 (3.3)	6 (10.9)	0.08
Pharyngeal hyperemia	6 (22.2)	23 (74.2)	29 (50.0)	<0.05
Pulmonary Infiltrates	22 (88.0)	19 (61.3)	41 (73.2)	<0.05
PPV and inotropic support	25 (92.6)	29 (93.5)	54 (93.1)	1.0
Capillary leak‡	21 (84.0)	18 (58.1)	39 (69.6)	<0.05
Admission laboratory findings§				
Thrombocyte count, × 10^3^/μL, mean (SD)	120.1 (81.6)	119.2 (76.9)	119.6 (78.4)	0.97
Abnormal coagulation tests, PT, PTT, INR, s, n = 49	24 (100)	23 (92.0)	47 (95.9)	0.49
Pyuria, n = 24	12 (50.0)	12 (50.0)	24 (50.0)	1.00
BUN, mg/dL, median (IQR), n = 57	14 (8–26)	13 (11–41)	14 (11–27)	0.73
Creatinine, g/day median (IQR)	0.8 (0.5–1.2)	0.9 (0.7–2.0)	0.8 (0.5–1.6)	0.12
eGFR, mL/min/m², mean (SD)	72.8 (34.2)	71.9 (39.8)	72.3 (37.0)	0.93
ALT, U/L, median (IQR)	49 (28–129)	40 (29–95)	47 (29–95)	0.55
AST, U/L, median (IQR)	52 (28–132)	52 (25–84)	52 (28–102)	0.62
Total bilirubin, md/dL, median (IQR), n = 57	0.9 (0.5–1.6)	0.9 (0.4–1.8)	0.9 (0.4–1.6)	0.79
CPK, U/L, median (IQR), n = 17	154 (73–674)	130 (67–304)	137 (69–304)	0.67
Treatment				
IVIg and clindamycin	14 (51.9%)	14 (45.2)	28 (48.3)	<0.05
Clindamycin only	8 (29.6%)	16 (51.6)	24 (41.4)	
IVIg only	0 (0.0%)	1 (3.2)	1 (1.7)	
Neither IVIg nor clindamycin	5 (18.5%)	0	5 (8.6)	

*ALT, alanine transaminase; AST, aspartate transaminase; BMI, body mass index; BUN, blood urea nitrogen; CPK, creatinine phosphokinase; eGFR, estimated glomerular filtration rate; INR, international normalized ratio; IQR, interquartile range; IVIg, intravenous immunoglobulin; NSTSS, nonstreptococcal TSS; PPV, positive pressure ventilation; PT, prothrombin time; PTT, partial thromboplastin time; STSS, streptococcal TSS; TSS, toxic shock syndrome.  †Continuous variables are presented as means with SDs; categorical variables are presented as counts with percentages. If data were not available for all patients, the number of patients is indicated. ‡Capillary leak indicates hypotension, hypoalbuminemia, and hemoconcentration. §Reference values: thrombocyte count, 140–440 × 10^3^/μL; PT, 12.4–14.7 s; PTT, 24–36 s; INR, <1.1; BUN, 5–18 mg/dL; creatinine, varies by patient age and sex; eGFR, >60 mL/min/1.73 m²; ALT, <40 U/L); AST, 15–50 U/L); bilirubin, 0.1–1.0 mg/dL; CPK, 37–430 U/L.

### Statistical Analysis

We compared the demographic, clinical, laboratory, and treatment data between patients with STSS and NSTSS. We used Student *t* tests to compare normally distributed continuous variables between STSS and NSTSS groups, the Wilcoxon rank-sum test to analyze skewed continuous variables, and a Fisher exact test to analyze categorical variables. Continuous variables are presented as means with SDs for normally distributed variables and as median and interquartile range for skewed variables; categorical variables are presented as counts with percentages.

We conducted all analyses on log-transformed values of the time from admission outcomes to satisfy model assumptions. We used Student *t* tests to compare STSS with NSTSS and a linear mixed model to compare groups of antimicrobial drugs. For ease of interpretation, raw means and SDs are presented for each group, and the mean difference of the log-transformed values are presented as the ratio of geometric means with 95% CI.

## Results

During the study period, 456 patients had diagnosis codes of TSS, severe sepsis with septic shock, GAS bacteremia, and necrotizing fasciitis at admission, discharge, or both. Our review of electronic medical records indicated that illnesses of 58 patients met the CDC diagnostic criteria for TSS. None of the patients had a prolonged preadmission course that would affect their treatment start time. Twenty-seven (47%) patients had STSS and 31 (53%) had NSTSS. Of the STSS patients, 16 had confirmed and 11 had probable STSS. Of the NSTSS patients, 11 had confirmed and 20 had probable NSTSS.

### Clinical and Laboratory Findings

Fever was not always documented during initial examinations, although most patients had history of fever requiring antipyretic medication or had fever develop during hospitalization (Table 1). Vomiting and rash were present in most patients with TSS. However, of the 27 patients with STSS, 13 (48%) had no rash. Altered mental status was observed in ≈40% of patients. 

Of patients with confirmed STSS, GAS was isolated from blood (3 patients), deep tissue (4 patients from necrotic tongue, subdural empyema, orbital abscess, and sinus aspirations), pleural fluid and deep endotracheal tube suction (4 patients), peritoneal fluid (1 patient), endotracheal tube suction and blood (3 patients), and urine and blood (1 patient). Patients with probable STSS had GAS obtained from rapid streptococcal screening test and throat cultures. Patients with NSTSS had staphylococci isolated from skin lesions and abscesses (10 patients); sterile sites, including bone (3 patients); urine (1 patient); and vaginal and genital area swab samples (7 patients).

Several patients with NSTSS, for which only thrombocytopenia is listed in the hematologic category of the CDC definition, had other coagulation abnormalities. Of 25 patients who had coagulation tests on admission, 17 (68%) had increased prothrombin time, 7 (28%) had increased prothrombin and partial thromboplastin time, and 1 patient had prolonged partial thromboplastin time. 

Inflammatory markers were not tested in all patients at admission. For the 20 patients for whom erythrocyte sedimentation rate (ESR) was tested on admission, median ESR was 22 mm/h (IQR 15.0–36.0 mm/h; reference 0–13 mm/h). ESR levels were higher in patients with STSS. Of 15 patients with NSTSS, median ESR was 22 mm/h (IQR 15.0–32.5 mm/h); of 5 patients with STSS, median ESR was 36 mm/h (IQR 14.5–39 mm/h). Of 34 patients for whom C-reactive protein (CRP) was tested on admission, median CRP was 12.75 mg/dL (IQR 6.8–23.8 mg/dL; reference <1.2 mg/dL). Of 18 patients with NSTSS, median CRP was 8.3 mg/dL (IQR 5.8–20.6 mg/dL), and of 16 patients with STSS, median CRP was 20.4 mg/dL (IQR 6.9–26.3mg/dL). CRP levels were significantly higher for patients with STSS than for those with NSTSS (p = 0.0443).

Pulmonary infiltrates were present in most (42 patients) patients with TSS at diagnosis and admission. Only 7 (12%) patients had primary admission and discharge diagnoses for pneumonia. Median arterial oxygen partial pressure to fractional inspired oxygen (PaO2:FiO2) ratio was also decreased in these patients. Of the 31 patients with NSTSS, for which pulmonary involvement is not a CDC diagnostic criterion, 19 (61%) had diffuse pulmonary infiltrates.

Of the clinical criteria listed for TSS and NSTSS and evaluated on admission, creatine phosphokinase was not elevated in the 17 patients for whom results were available. Similarly, renal and hepatic involvement criteria for both STSS and NSTSS did not vary significantly from age-based normal values for most of the patients. Twelve (21%) patients had elevated alanine transaminase, 14 (24%) had elevated aspartate transaminase, and 17 (29%) had elevated serum creatinine. However, pyuria (>10 leukocytes/high-power field), which is not a CDC clinical criterion for STSS, was present in half the patients with STSS who had a urinalysis on admission (median urine leukocyte count 17.5/high-power field). We also reviewed microbiological studies. For 21 (68%) patients with NSTSS, *Staphylococcus aureus* grew from superficial and sterile site cultures.

We found significant differences between patients with STSS and NSTSS. Patients with STSS were significantly younger, had lower body mass index, were less likely to have an erythematous rash, and had more capillary leak and pulmonary infiltrates at presentation. Patients with NSTSS were less likely to have pulmonary infiltrates and capillary leak and to have less tissue necrosis or mental status changes (Table 1).

### Management of Patients

Patients with NSTSS received clindamycin and IVIg sooner than patients with STSS. Five patients who did not receive clindamycin or IVIg had STSS. Patients with NSTSS stayed ≈57% fewer inpatient days (Tables 1, 2; Appendix). One patient had necrotizing infection of the tongue that was promptly recognized and operatively debrided. One patient with STSS died. We examined the differences between patients receiving only clindamycin, only IVIg, both, or neither. None of the outcomes differed significantly among groups (Appendix). 

**Table 2:** Time from hospital admission for patients who had streptococcal toxic shock syndrome with and without rash and treatments received, Nationwide Children’s Hospital, Columbus, Ohio, USA, 2010–2017

Time from admission, d	Rash, mean (± SD)	No rash, mean (± SD)	Ratio of geometric means (95% CI)	p value
Start of antimicrobial drugs	0.45 (0.49)	1.08 (1.57)	0.47 (0.16–1.40)	0.17
Start of clindamycin	0.87 (0.61)	3.37 (2.36)	0.24 (0.10–0.59)	<0.05
Start of intravenous immunoglobulin	1.47 (0.60)	1.48 (0.49)	0.97 (0.58–1.60)	0.89

Management of STSS patients with rash differed from management of those without rash. We found no difference in the requirement for vasopressor support between patients with or without rash, yet clindamycin was initiated ≈66% sooner for patients with rash than for patients without rash (Table 2). Patients with STSS and without a rash had longer hospital stays than did those with rash. Clindamycin with or without IVIg infusion was delayed in treatment of STSS patients without rash, and for 5 patients (19% of patients without rash), neither clindamycin nor IVIg was administered.

## Discussion

Data are limited on the incidence and management of children with TSS (*3*,*8*). Our large, single-institution retrospective analysis of 58 cases showed similarities and unexpected findings among children with TSS. In comparison with patients with NSTSS, children with STSS were younger, experienced more severe illness with higher rates of capillary leak and pulmonary symptoms, and had longer hospital stays. Similar to our findings, STSS has been associated with more severe disease and worse outcomes than NSTSS in the literature (*2*,*3*,*7*,*8*,*15*,*17*). Unlike our findings, those studies do not indicate whether late recognition and treatment of STSS in patients without rash played any role in the reported disease outcomes and hospitalization duration.

Case definitions for streptococcal and NSTSS are generally designed to optimize specificity rather than sensitivity. Our data suggest that unified criteria for TSS, rather than separate criteria for STSS and NSTSS, might improve sensitivity. We found that certain clinical and laboratory findings were common in both STSS and NSTSS but only included in the case definition of one or the other. First, although pyuria is a criterion for NSTSS but not for STSS (*4*,*5*), half the urinalyses performed in both groups demonstrated pyuria. Second, acute respiratory distress syndrome is a criterion for STSS but not for NSTSS. However, 61% of the patients in our study with NSTSS had diffuse pulmonary infiltrates and decreased PaO2:FiO2 ratio <200. Finally, coagulation abnormalities are included in the case definition of STSS but not NSTSS, yet most patients in both groups had abnormalities. The pulmonary involvement, pyuria, and coagulation abnormalities were common and were observed at admission. Case definitions are generally designed to have high specificity, sometimes at the expense of sensitivity. If the relatively inexpensive evaluation methods, such as urinalysis, chest radiograph, PaO2:FiO2 ratio of <200 and coagulation panels were incorporated into TSS evaluations, our data suggest that they might have aided accurate and timely diagnosis. Consideration of these additional organ dysfunctions, and considering TSS as a unifying diagnosis rather than as STSS and NSTSS, could potentially strengthen the conclusive diagnosis rather than discrediting it.

Data from published literature illustrate the importance of prompt recognition of TSS and early treatment (*7*,*18*–*24*). Complementary treatment with clindamycin improves the survival of patients with STSS by reducing toxin release (*7*,*18*–*24*). Although IVIg is suggested as a possible adjunctive agent, published patient outcomes have varied (*24*–*28*). IVIg functions by inhibiting T-cell activation that would result in decreased cytokine release, down-regulation of adhesion molecules, chemokine and chemokine-receptor expression, and neutralization of superantigens (*25*–*28*). In some studies, IVIg did not show significant benefit in treatment of patients with STSS (*20*,*24*,*29*). One report that pooled 5 studies of clindamycin and IVIg treatment for STSS showed the death rates decreased from 33.7% to 15.7% (*30*). We did not identify significant differences in hospitalization rates and duration of mechanical ventilation among patients treated with or without IVIg. Fewer than half the patients in our study received clindamycin and IVIg. Similarly, only 50%–67% of patients with severe GAS infection have received clindamycin (*2*,*18*,*20*,*24*). Our results suggest that the lack of appropriate treatment may be associated with absence of rash at the time of presentation. In our study, in STSS patients without rash, sepsis alone was often diagnosed, and the patients were not treated with clindamycin or IVIg. These patients also experienced delays in diagnosis and had longer hospital stays than those with a rash.

Our study had several limitations. Although Nationwide Children’s Hospital serves almost all of central Ohio’s children, our data were not population based and were from 1 pediatric center. We might not have identified all patients with NSTSS because we were unable to review individual charts for patients with staphylococcal bacteremia if they did not have additional diagnoses of sepsis or septic shock. Despite our efforts to capture the clinical and laboratory changes at the time of initial evaluation, the duration of illness, as well as rapidly changing signs such as rash, might not have been accurately recorded in the patient charts, which would affect the overall diagnosis and possibly lead to misclassification of patients. In addition, because of the retrospective nature of our study, none of the bacterial isolates were archived for further typing or microbiological analysis.

Our findings indicate organ system involvement in TSS beyond that predicted by existing CDC criteria. Because clinicians appear to rely on archetypal presentations of TSS (e.g., fever, hypotension, and rash) to diagnose TSS, updated clinical and diagnostic criteria would facilitate early accurate diagnosis and treatment. Physicians should be aware of TSS presentations without rash. Furthermore, the updated criteria may benefit from a unifying definition.

Appendix Additional results for study of manifestations of toxic shock syndrome in children, Columbus, Ohio, USA.

## References

[1] Stevens DL, Tanner MH, Winship J, Swarts R, Ries KM, Schlievert PM, et al. Severe group A streptococcal infections associated with a toxic shock-like syndrome and scarlet fever toxin A. N Engl J Med. 1989;321:1–7. 10.1056/NEJM198907063210101 2659990

[2] Adalat S, Dawson T, Hackett SJ, Clark JE; In association with the British Paediatric Surveillance Unit. Toxic shock syndrome surveillance in UK children. Arch Dis Child. 2014;99:1078–82. 10.1136/archdischild-2013-304741 24790135

[3] Javouhey E, Bolze PA, Jamen C, Lina G, Badiou C, Poyart C, et al. Similarities and differences between staphylococcal and streptococcal toxic shock syndromes in children: results from a 30-case cohort. Front Pediatr. 2018;6:360. 10.3389/fped.2018.00360 30547021 PMC6280580

[4] Centers for Disease Control and Prevention. Streptococcal toxic shock syndrome (STSS) (*Streptococcus pyogenes*) 2010 case definition [cited 2019 May 23]. https://wwwn.cdc.gov/nndss/conditions/streptococcal-toxic-shock-syndrome/case-definition/2010

[5] Centers for Disease Control and Prevention. Toxic shock syndrome (other than streptococcal) (TSS) 2011 case definition [cited 2019 May 23]. https://wwwn.cdc.gov/nndss/conditions/toxic-shock-syndrome-other-than-streptococcal/case-definition/2011

[6] O’Brien KL, Beall B, Barrett NL, Cieslak PR, Reingold A, Farley MM, et al. Epidemiology of invasive group a streptococcus disease in the United States, 1995-1999. Clin Infect Dis. 2002;35:268–76. 10.1086/341409 12115092

[7] Chen KY, Cheung M, Burgner DP, Curtis N. Toxic shock syndrome in Australian children. Arch Dis Child. 2016;101:736–40. 10.1136/archdischild-2015-310121 27117838

[8] Chuang YY, Huang YC, Lin TY. Toxic shock syndrome in children: epidemiology, pathogenesis, and management. Paediatr Drugs. 2005;7:11–25. 10.2165/00148581-200507010-00002 15777108

[9] Nelson GE, Pondo T, Toews KA, Farley MM, Lindegren ML, Lynfield R, et al. Epidemiology of invasive group A streptococcal infections in the United States, 2005–2012. Clin Infect Dis. 2016;63:478–86. 10.1093/cid/ciw248 27105747 PMC5776658

[10] Tyrrell GJ, Fathima S, Kakulphimp J, Bell C. Increasing rates of invasive group A streptococcal disease in Alberta, Canada; 2003–2017. Open Forum Infect Dis. 2018;5:ofy177. 10.1093/ofid/ofy177 30109241 PMC6084600

[11] Davies HD, Matlow A, Scriver SR, Schlievert P, Lovgren M, Talbot JA, et al. Apparent lower rates of streptococcal toxic shock syndrome and lower mortality in children with invasive group A streptococcal infections compared with adults. Pediatr Infect Dis J. 1994;13:49–56. 10.1097/00006454-199401000-00011 8170732

[12] Descloux E, Perpoint T, Ferry T, Lina G, Bes M, Vandenesch F, et al. One in five mortality in non-menstrual toxic shock syndrome versus no mortality in menstrual cases in a balanced French series of 55 cases. Eur J Clin Microbiol Infect Dis. 2008;27:37–43. 10.1007/s10096-007-0405-2 17932694

[13] Rodríguez-Nuñez A, Dosil-Gallardo S, Jordan I; ad hoc Streptococcal Toxic Shock Syndrome collaborative group of Spanish Society of Pediatric Intensive Care. Clinical characteristics of children with group A streptococcal toxic shock syndrome admitted to pediatric intensive care units. Eur J Pediatr. 2011;170:639–44. 10.1007/s00431-010-1337-x 20981441

[14] Shah SS, Hall M, Srivastava R, Subramony A, Levin JE. Intravenous immunoglobulin in children with streptococcal toxic shock syndrome. Clin Infect Dis. 2009;49:1369–76. 10.1086/606048 19788359 PMC2761980

[15] Timmis A, Parkins K, Kustos I, Riordan FA, Efstratiou A, Carrol ED. Invasive group A streptococcal infections in children presenting to a paediatric intensive care unit in the North West of England. J Infect. 2010;60:183–6. 10.1016/j.jinf.2009.12.001 20004215

[16] Lin YJ, Cheng MC, Lo MH, Chien SJ. Early differentiation of Kawasaki disease shock syndrome and toxic shock syndrome in a pediatric intensive care unit. Pediatr Infect Dis J. 2015;34:1163–7. 10.1097/INF.0000000000000852 26222065

[17] Lithgow A, Duke T, Steer A, Smeesters PR. Severe group A streptococcal infections in a paediatric intensive care unit. J Paediatr Child Health. 2014;50:687–92. 10.1111/jpc.12601 24909187

[18] Linnér A, Darenberg J, Sjölin J, Henriques-Normark B, Norrby-Teglund A. Clinical efficacy of polyspecific intravenous immunoglobulin therapy in patients with streptococcal toxic shock syndrome: a comparative observational study. Clin Infect Dis. 2014;59:851–7. 10.1093/cid/ciu449 24928291

[19] Andreoni F, Zürcher C, Tarnutzer A, Schilcher K, Neff A, Keller N, et al. Clindamycin affects group A streptococcus virulence factors and improves clinical outcome. J Infect Dis. 2017;215:269–77. 27247345 10.1093/infdis/jiw229

[20] Couture-Cossette A, Carignan A, Mercier A, Desruisseaux C, Valiquette L, Pépin J. Secular trends in incidence of invasive beta-hemolytic streptococci and efficacy of adjunctive therapy in Quebec, Canada, 1996-2016. PLoS One. 2018;13:e0206289. 10.1371/journal.pone.0206289 30352091 PMC6198987

[21] Stevens DL, Gibbons AE, Bergstrom R, Winn V. The Eagle effect revisited: efficacy of clindamycin, erythromycin, and penicillin in the treatment of streptococcal myositis. J Infect Dis. 1988;158:23–8. 10.1093/infdis/158.1.23 3292661

[22] Sriskandan S, McKee A, Hall L, Cohen J. Comparative effects of clindamycin and ampicillin on superantigenic activity of *Streptococcus pyogenes.* J Antimicrob Chemother. 1997;40:275–7. 10.1093/jac/40.2.275 9301995

[23] Zimbelman J, Palmer A, Todd J. Improved outcome of clindamycin compared with beta-lactam antibiotic treatment for invasive *Streptococcus pyogenes* infection. Pediatr Infect Dis J. 1999;18:1096–100. 10.1097/00006454-199912000-00014 10608632

[24] Carapetis JR, Jacoby P, Carville K, Ang SJ, Curtis N, Andrews R. Effectiveness of clindamycin and intravenous immunoglobulin, and risk of disease in contacts, in invasive group a streptococcal infections. Clin Infect Dis. 2014;59:358–65. 10.1093/cid/ciu304 24785239

[25] Norrby-Teglund A, Muller MP, Mcgeer A, Gan BS, Guru V, Bohnen J, et al. Successful management of severe group A streptococcal soft tissue infections using an aggressive medical regimen including intravenous polyspecific immunoglobulin together with a conservative surgical approach. Scand J Infect Dis. 2005;37:166–72. 10.1080/00365540410020866 15849047

[26] Schlievert PM. Use of intravenous immunoglobulin in the treatment of staphylococcal and streptococcal toxic shock syndromes and related illnesses. J Allergy Clin Immunol. 2001;108(Suppl):S107–10. 10.1067/mai.2001.117820 11586276

[27] Valiquette L, Low DE, McGeer AJ. Assessing the impact of intravenous immunoglobulin in the management of streptococcal toxic shock syndrome: a noble but difficult quest. Clin Infect Dis. 2009;49:1377–9. 10.1086/606049 19788361

[28] Low DE. Toxic shock syndrome: major advances in pathogenesis, but not treatment. Crit Care Clin. 2013;29:651–75. 10.1016/j.ccc.2013.03.012 23830657

[29] Kadri SS, Swihart BJ, Bonne SL, Hohmann SF, Hennessy LV, Louras P, et al. Impact of intravenous immunoglobulin on survival in necrotizing fasciitis with vasopressor-dependent shock: a propensity score-matched analysis from 130 US hospitals. Clin Infect Dis. 2017;64:877–85. 28034881 10.1093/cid/ciw871 PMC5850528

[30] Parks T, Wilson C, Curtis N, Norrby-Teglund A, Sriskandan S. Polyspecific intravenous immunoglobulin in clindamycin-treated patients with streptococcal toxic shock syndrome: a systematic review and meta-analysis. Clin Infect Dis. 2018;67:1434–6. 10.1093/cid/ciy401 29788397 PMC6186853

