# Supplementary material for: Manifestations of Toxic Shock Syndrome in Children, Columbus, Ohio, USA, 2010–2017
Source: Emerg Infect Dis. 2020 Jun;26(6):1077–83. doi: 10.3201/eid2606.190783 (PMC7258457; doi:10.3201/eid2606.190783)
Supplement: Appendix — Additional results for study of manifestations of toxic shock syndrome in children, Columbus, Ohio, USA. [file 19-0783-Techapp-s1.pdf]

Article DOI: https://doi.org/10.3201/eid2606.190783

Manifestations of Toxic Shock Syndrome in Children, Columbus, Ohio, USA, 2010–2017

Appendix

Appendix Table 1. Comparisons for DFA variables for children with TSS, Columbus, Ohio, USA, 2010–2017

Variable	STSS	NSTSS	Ratio of geometric means (95% CI)	p value
Inpatient days	16.54 (12.60)	7.71 (9.58)	0.43 (0.29–0.63)	<0.05
DFA to first bolus	0.40 (0.39)	0.34 (0.98)	0.45 (0.18–1.14)	0.09
DFA to start of antimicrobial drugs†	0.75 (1.17)	0.33 (0.42)	0.47 (0.23–0.93)	0.03
DFA to clindamycin	1.89 (1.98)	0.44 (0.42)	0.27 (0.15–0.50)	<0.05
DFA to IVIg start	1.47 (0.55)	1.06 (1.17)	0.47 (0.26–0.87)	<0.05
DFA to vasopressor start‡	0.57 (0.51)	0.48 (0.97)	0.49 (0.20–1.23)	0.12
DFA to TSS diagnosis	2.39 (1.91)	1.41 (1.24)	0.52 (0.27–1.01)	0.05
Total days on vasopressors	2.48 (1.95)	2.42 (4.29)	0.76 (0.36–1.60)	0.46

*Continuous variables are presented as means (± SD). DFA, days from admission; IVIg, intravenous immunoglobulin; NSTSS, nonstreptococcal TSS; STSS, streptococcal TSS; TSS, toxic shock syndrome.
†Excludes clindamycin.
‡Epinephrine, norepinephrine, dopamine, milrinone.

Appendix Table 2. Comparisons for DFA variables and treatments for children with TSS, Columbus, Ohio, USA, 2010–2017*

Variable	Clindamycin and IVIg	Clindamycin only	IVIg only†	Neither	p value
Inpatient days	14.15 (13.54)	8.77 (9.77)	4.83	14.75 (9.90)	0.12
DFA to TSS diagnosis	1.89 (1.60)	1.69 (1.59)	0.72	NA	0.52
DFA to first intravenous fluid bolus	0.30 (0.36)	0.49 (1.12)	0.003	0.22 (0.29)	0.25
DFA to vasopressor start	0.67 (0.95)	0.33 (0.32)	0.14	0.18 (0.11)	0.80
Total days on vasopressor support	3.15 (4.15)	1.33 (0.66)	1.46	1.25 (0.63)	0.75

*Continuous variables are presented as means (± SD). DFA, days from admission; IVIg, intravenous immunoglobulin; NA, no data available; TSS, toxic shock syndrome.
†Because measurements from only 1 patient were available, SDs could not be calculated.

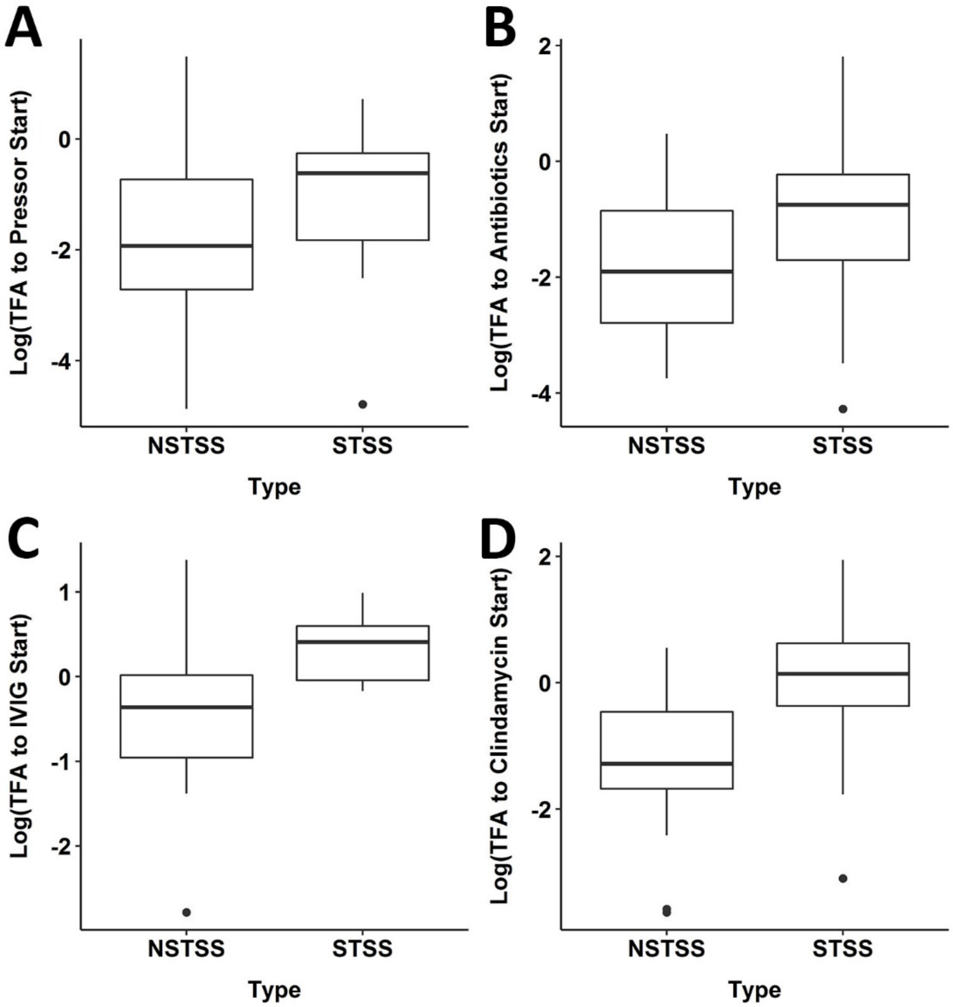

Appendix Figure. Boxplots demonstrating that TFA to starting to pressors, antimicrobial drugs, IVIG, and clindamycin were all higher for STSS patients than for NSTSS patients. These differences are statistically significant for TFA to starting antimicrobial drugs, IVIG, and clindamycin, indicating that the STSS patients in this study typically received these treatments later than did NSTSS patients. IVIG, intravenous immunoglobulin; NSTSS, nonstreptococcal TSS; STSS, streptococcal TSS; TFA, time from admission (days); TSS, toxic shock syndrome.
